# Supplementary material for: Using long-read sequencing to detect imprinted DNA methylation
Source: Nucleic Acids Res. 2019 Feb 22;47(8):e46. doi: 10.1093/nar/gkz107 (PMC6486641; doi:10.1093/nar/gkz107)
Supplement: Supplementary Data [file gkz107_supplemental_files.zip › SuppFigures.pdf]

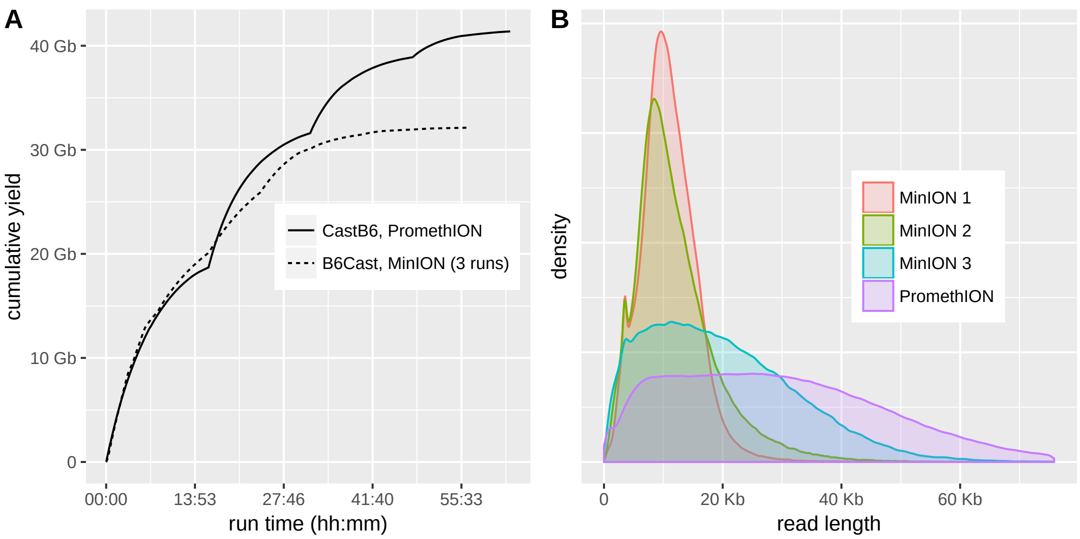

Figure S1. Nanopore sequencing yield and read length. The B6 × Cast (B6Cast) F1 sample was sequenced on three MinION flowcells, and the Cast × B6 (CastB6) F1 on one PromethION flowcell. **A.** Yield of sequencing runs over time (the three MinION runs are merged). **B.** Read length distribution in each sequencing run.

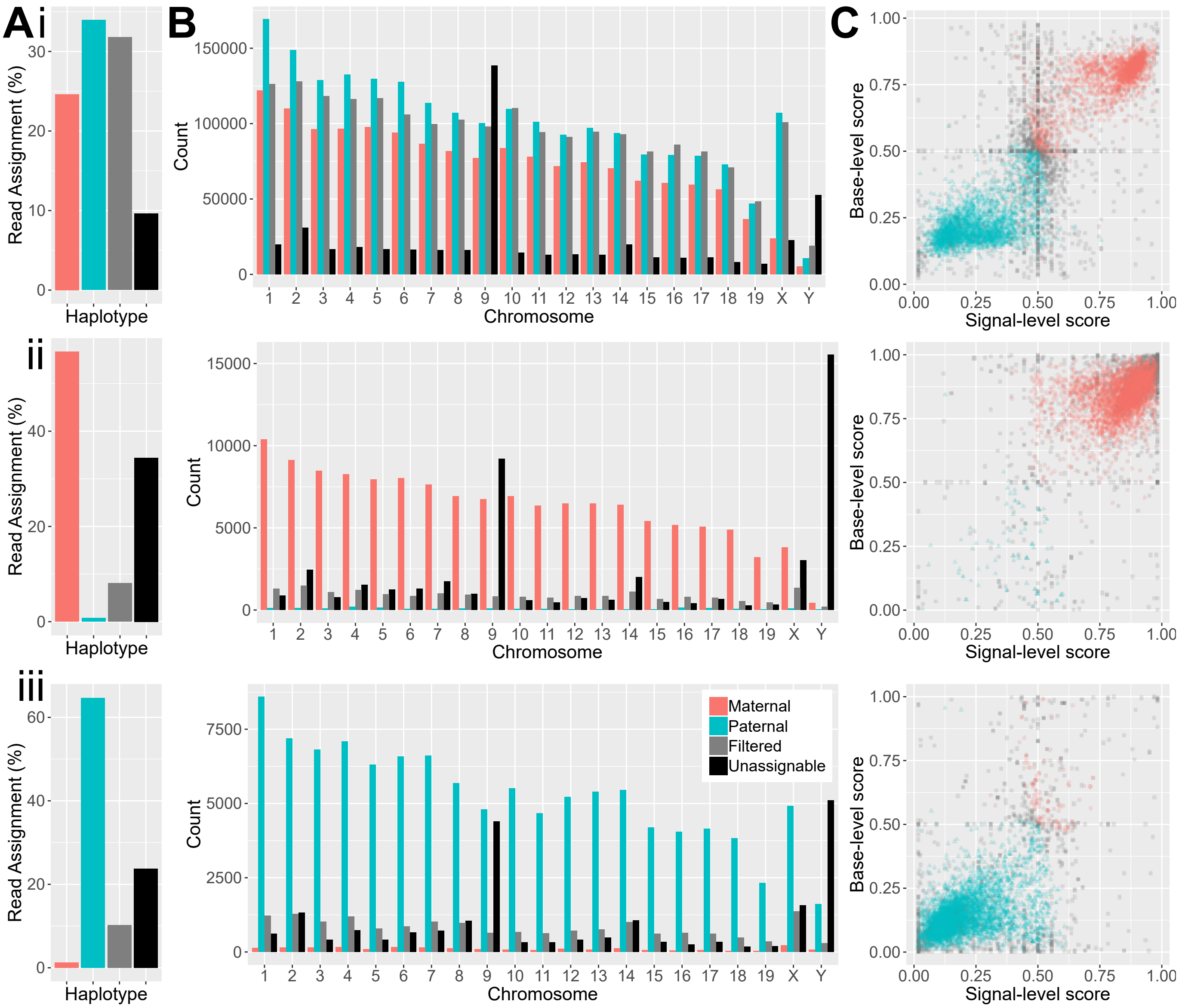

Figure S2. Haplotyping of the Cast × B6, B6 and Cast samples. A. Percentages of mapped reads from nanopore sequencing that were assigned to the B6 genome (maternal), Cast genome (paternal), or that could not be haplotyped (filtered) for the Cast × B6 F1 sample (i), $B6$ F0 sample (ii) and Cast F0 sample (iii). **B.** Percentages of mapped reads from nanopore sequencing of each sample that were assigned to each haplotype on each chromosome. **C.** Scatter plot of haplotype scores for nanopore reads according to signal (x-axis) and basecall (y-axis) methods. Only 10,000 randomly selected reads are shown for ease of visualisation.

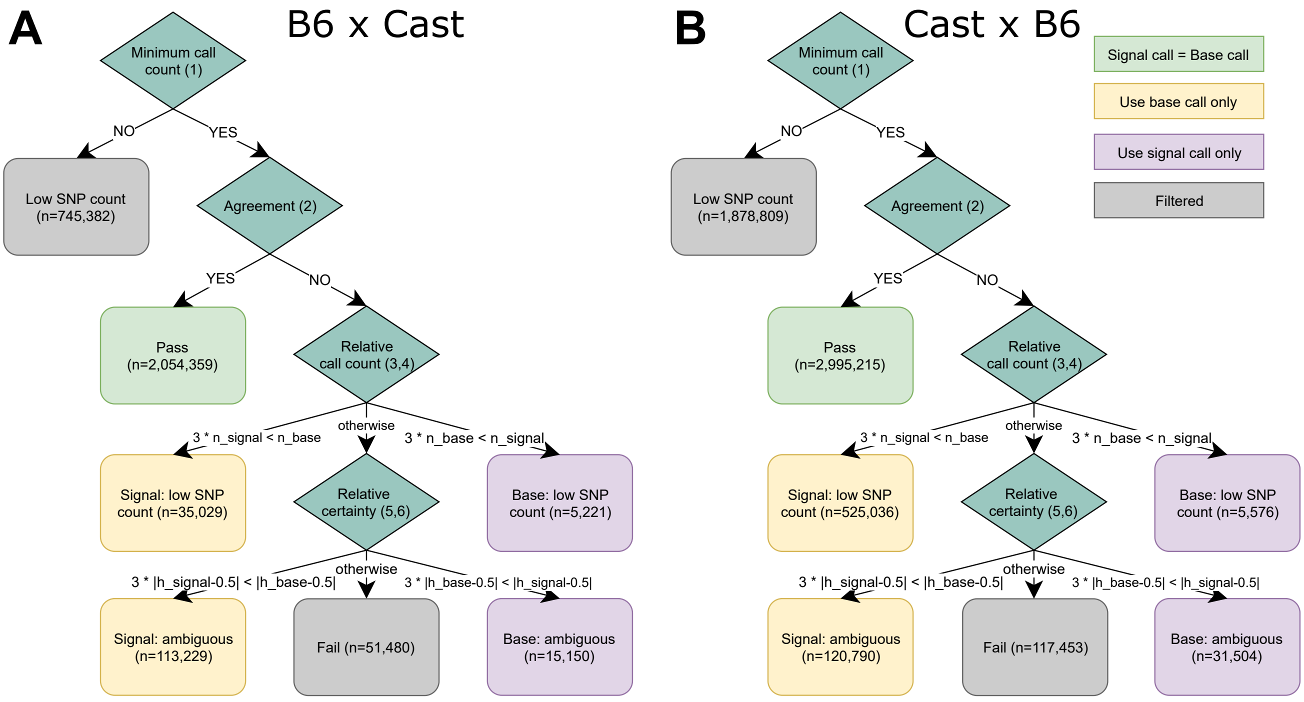

Figure S3. Haplotyping flow chart. A. Haplotyping flow chart for B6 $\times$ Cast forward cross MinION data. **B.** Haplotyping flow chart for Cast $\times$ B6 reverse cross PromethION data.

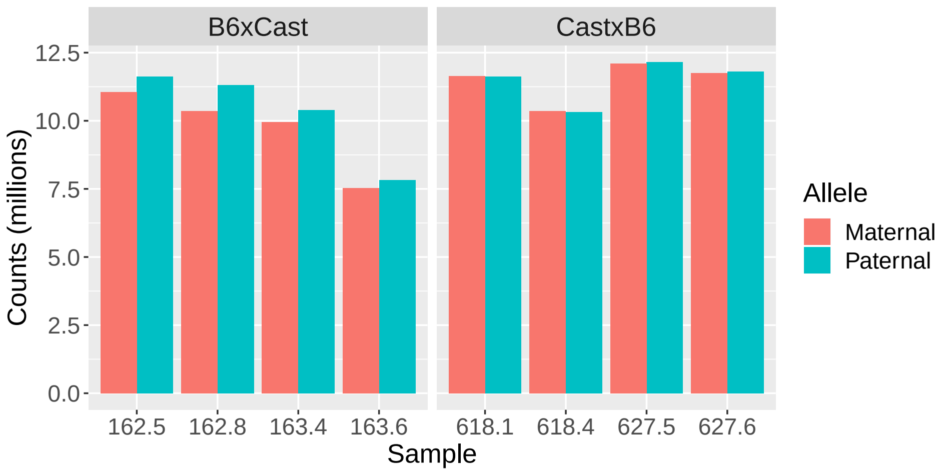

Figure S4. RNA-seq library sizes by allele. Consistent paternal and maternal counts indicate that there was no contamination by maternal tissue. The slightly lower maternal counts in the B6Cast samples are due to the FVB regions in the maternal strain (see Methods).

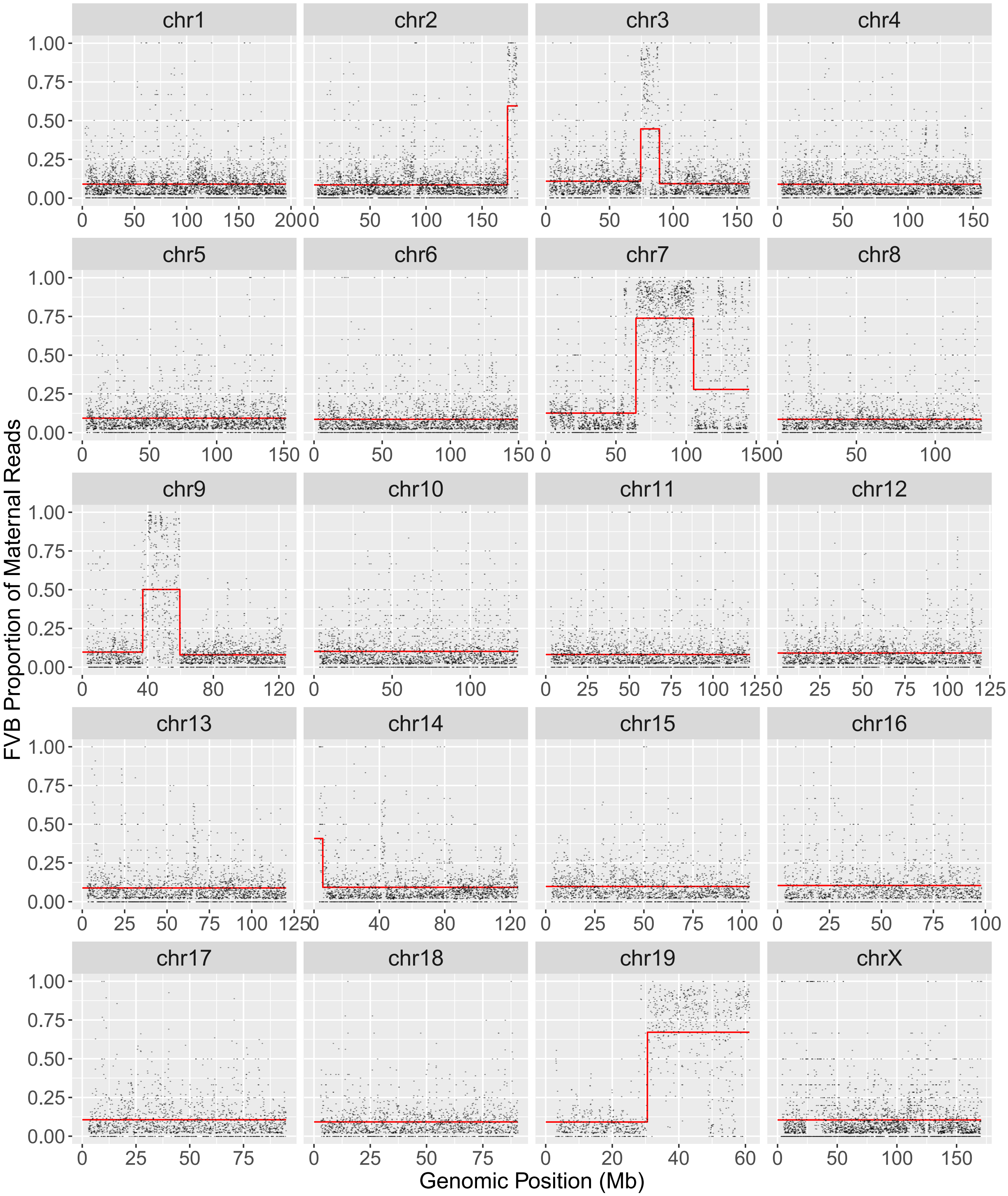

Figure S5. Recursive partition tree resolution of FVB genotype. Proportion of maternal reads from the B6xCast sample assigned to the FVB genotype by genomic location, where the remainder of the maternal reads are assigned to the C57BL/6 reference genotype. Sections of the genome where the regression tree gave a value higher than 0.4 (shown in red) were assigned to the FVB genotype. The Y chromosome is excluded.
